# Study of GaN coalescence by dark-field X-ray microscopy at the nanoscale

**DOI:** 10.1107/S160057672300287X

**Published:** 2023-04-25

**Authors:** Maya Wehbe, Matthew Charles, Kilian Baril, Blandine Alloing, Daniel Pino Munoz, Nabil Labchir, Jesús Zuniga-Perez, Carsten Detlefs, Can Yildirim, Patrice Gergaud

**Affiliations:** a Univ. Grenoble Alpes, CEA-LETI, 38000 Grenoble, France; b MINES Paris, PSL Research University, CEMEF – Center for Material Forming, CNRS UMR 7635, BP 207, 1 rue Claude Daunesse, 06904 Sophia Antipolis Cedex, France; c Univ. Côte d’Azur, CNRS, CRHEA, Rue Bernard Gregory, 06560 Valbonne, France; d Univ. Grenoble Alpes, CNRS, CEA/LETI-Minatec, Grenoble INP, LTM, F-38054 Grenoble, France; e MajuLab, International Research Laboratory IRL 3654, CNRS, Université Côte d’Azûr, Sorbonne Université, National University of Singapore, Nanyang Technological University, Singapore, Singapore; f ESRF, The European Synchrotron, 71 Avenue des Martyrs, CS40220, 38043 Grenoble Cedex 9, France; Montanuniversität Leoben, Austria

**Keywords:** dark-field X-ray microscopy, synchrotron radiation, characterization, gallium nitride, coalescence, diffraction imaging, nano-pillars

## Abstract

This work illustrates the potential of dark-field X-ray microscopy (DFXM), a 3D imaging technique of nanostructures, in characterizing novel epitaxial structures of gallium nitride (GaN) on top of GaN/AlN/Si/SiO_2_ nano-pillars for optoelectronic applications. The nano-pillars are intended to allow independent GaN nanostructures to coalesce into a highly oriented film due to the SiO_2_ layer becoming soft at the GaN growth temperature. DFXM is demonstrated on different types of samples at the nanoscale and the results show that extremely well oriented lines of GaN (standard deviation of 0.04°) as well as highly oriented material for zones up to 10 × 10 µm^2^ in area are achieved with this growth approach. At a macroscale, high-intensity X-ray diffraction is used to show that the coalescence of GaN pyramids causes misorientation of the silicon in the nano-pillars, implying that the growth occurs as intended (*i.e.* that pillars rotate during coalescence). These two diffraction methods demonstrate the great promise of this growth approach for micro-displays and micro-LEDs, which require small islands of high-quality GaN material, and offer a new way to enrich the fundamental understanding of optoelectronically relevant materials at the highest spatial resolution.

## Abstract 2

In this article, highly oriented small structures of gallium nitride grown on top of silicon nano-pillars are characterized by dark-field X-ray microscopy for optoelectronic applications such as micro-LEDs.

## 1. Introduction

X-ray diffraction is a powerful characterization technique used to assess the crystallinity of materials by determining the orientation of crystalline regions, measuring the stress present in films and even resolving their atomic structure. Over the years, many diffraction-based 3D mapping approaches have been developed and used, but they have several limitations; for example, electron-microscopy methods (Liu *et al.*, 2011 ▸) are destructive, and 3D X-ray diffraction (Poulsen *et al.*, 2001 ▸; Poulsen, 2004 ▸) and diffraction contrast tomography (Ludwig *et al.*, 2009 ▸) are restricted by the detector to a spatial resolution of 1 µm. However, dark-field X-ray microscopy (DFXM) is non-destructive (Yildirim *et al.*, 2021 ▸), is very well suited to simultaneous multiscale characterization (Simons *et al.*, 2015 ▸) or *in situ* measurements (Yildirim *et al.*, 2021 ▸), and can achieve direct spatial resolutions of 30–100 nm (Kutsal *et al.*, 2019 ▸) and a time resolution of 100 ms (Holstad *et al.*, 2022 ▸). The application of this technique has been largely demonstrated for metallurgical studies (Maziarz *et al.*, 2010 ▸), fuel cells (Longo *et al.*, 2020 ▸) and biominerals (Cook *et al.*, 2018 ▸), and it is only rarely performed on microelectronics devices (Jakobsen *et al.*, 2019 ▸). It is, however, a particularly interesting technique for microelectronic objects because it makes it possible to observe the individual behavior of a large number of objects, which can range from several hundred to several thousand (Yildirim *et al.*, 2021 ▸). In this way, it is therefore possible to detect phenomena such as defects or random growth. Among the other particularities of this technique is its ability to provide 3D mapping of strain and orientations in crystalline materials at the nanoscale and to generate a real-space image of the illuminated volume. The European Synchrotron Radiation Facility (ESRF) provides advanced techniques to study matter at the nanoscale with DFXM at the ID06-HXM beamline (Yildirim *et al.*, 2021 ▸). The energy ranges from 11 to 55 keV with a minimum beam size of 30.0 × 0.5 µm^2^ (*h* × *v*) and a maximum beam size of 2.0 × 0.5 mm^2^ (*h* × *v*). A compound refractive lens (CRL) is placed between the sample and a 2D detector to provide a magnified Bragg-diffracted beam. This method is able to perform both mosaicity scans and strain scans to construct a high-resolution reciprocal map of the studied volume.

In this work, we show that DFXM is extremely beneficial for optoelectronics applications at the nanoscale by characterizing structures of gallium nitride (GaN) grown on top of GaN/AlN/Si/SiO_2_ nano-pillars by a novel heteroepitaxial method. GaN is an extremely attractive semiconductor for optoelectronics applications (Miyajima *et al.*, 2001 ▸) such as virtual reality, augmented reality, smart watches *etc.*, due to its direct band gap and the possibility of emission in the 400–625 nm range when using InGaN alloy. This allows the fabrication of highly efficient light-emitting diodes (LEDs) from ultraviolet to blue, green and even red wavelengths (Dussaigne *et al.*, 2021 ▸). Epitaxial growth of GaN is very expensive due to the cost of substrates and limitations in size. Thus, growth of GaN on low-cost foreign substrates (sapphire, silicon and silicon carbide) is common. However, heteroepitaxy results in the generation of defects such as dislocations in the GaN layers of the order of 5 × 10^8^ cm^−2^ for GaN on silicon (Si) (Zhu *et al.*, 2012 ▸) and 4 × 10^8^ cm^−2^ for GaN on sapphire (Shen *et al.*, 2005 ▸), and this is mainly due to the large lattice parameter mismatch. Dislocations are responsible for degradation in the emission efficiency of GaN-based devices. Also, a large thermal-coefficient mismatch between the film and the substrate generates stress in the epilayer during the cooling phase of the growth process, potentially resulting in cracks and/or a wafer bow (Liu & Edgar, 2002 ▸). Various methods aiming to produce high-quality heteroepitaxial GaN with reduced dislocation density have been implemented, such as epitaxial lateral overgrowth (Dassonneville *et al.*, 2001 ▸) and selective-area growth (Tanaka *et al.*, 2017 ▸, 2000 ▸). However, these growth approaches do not address the issue of dislocation generation during the coalescence of neighboring GaN nuclei. When two nearby crystallites come into contact with one another from independent nucleation sites, they will inherit the crystallographic misalignment associated with the heteroepitaxial growth, as demonstrated by Mante *et al.* (2018 ▸) for aluminium nitride (AlN) grown on Si. As a result, coalescence boundary dislocations will be generated to accommodate this misorientation, leading to threading dislocations which will traverse the epilayer through the active region and reduce the performance of the GaN-based device. To tackle this issue of crystallite misorientation, we developed a novel heteroepitaxial approach that consists of growing GaN pyramids on top of nano-patterned GaN/AlN/Si(111)/SiO_2_ pillars on an Si(001) substrate. At high temperatures, the viscoelastic properties of SiO_2_ will enable a rotation or tilt of the pillars, which should allow the GaN on top to coalesce without the formation of grain boundaries. The dislocation densities in the GaN platelets obtained so far are in the low 10^8^ cm^−2^ (Dagher *et al.*, 2019 ▸).

DFXM provided us with the necessary structural properties of these coalesced GaN platelets at a high accuracy and high spatial resolution to determine their quality and optimize this novel growth method. We also studied the samples on the macroscale with the high-intensity X-ray diffraction technique at the BM02 beamline (Chahine *et al.*, 2019 ▸) to measure the degree of twist and tilt of the nano-pillars in the Si layer. This non-destructive technique is characterized by a wide energy range from 6 to 45 keV with a minimum beam size of 30.0 × 30 µm^2^ (*h* × *v*), a maximum beam size of 5 × 5 mm^2^ (*h* × *v*) (Chahine *et al.*, 2019 ▸) and high-brilliance X-rays.

In the following, we first present a detailed description of the studied samples and then we explain the two experiments conducted at ESRF. The last part is dedicated to presenting the results followed by a detailed discussion and a conclusion.

## 2. Materials and methods

### 2.1. Sample description

**Figure 1:**
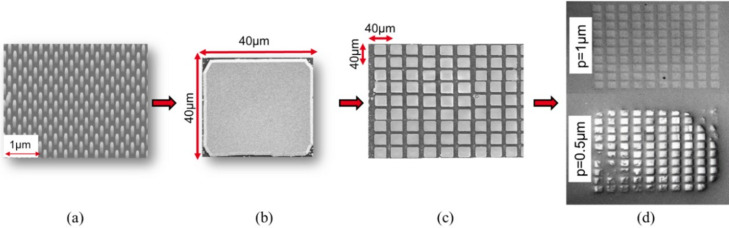
Scannning electron microscope images of (*a*) GaN/AlN/Si(111)/SiO_2_/Si(100) nano-pillars before GaN pyramid growth, (*b*) a single fully coalesced 40 × 40 µm^2^ GaN platelet, (*c*) a matrix of fully coalesced GaN platelets, and (*d*) two GaN matrices with structures coalesced on different pitch arrays (*p* = 0.5 µm and *p* = 1 µm).

In Fig. 1 ▸(*a*) we show a GaN/AlN/Si(111)/SiO_2_/Si(100) etched nano-pillar array with hexagonal symmetry and a pitch *p* (center-to-center distance between two consecutive pillars) of 500 nm. The buffer GaN layer, the AlN nucleation layer and the Si(111) layer have thicknesses of 250, 150 and 50 nm, respectively, while the SiO_2_ is etched down to a depth of 300 nm. The base substrate is Si(100), allowing us to perform diffraction only on the top Si layers, which display a different crystallographic orientation. The etching process of these 100 nm diameter nano-pillars has been previously explained in detail by Mrad *et al.* (2022 ▸). The next step for fabricating GaN platelets is the growth of GaN pyramids on top of these nano-pillars by metal organic vapor phase epitaxy, with tri­methyl­gallium (TMGa) and ammonia (NH_3_) as precursors for gallium and nitro­gen, respectively. At high-growth temperatures (∼1323.15 K), the viscoelastic properties of SiO_2_ facilitate its deformation, potentially enabling a rotation or tilt of the pillars, which should allow the GaN pyramids to align as they join and prevent the formation of grain boundaries. This alignment is driven by the excess energy present at the interfaces between the pyramids and should result in the formation of a low threading dislocation density GaN platelet. Then, a 2D GaN growth step is performed to obtain a 40 × 40 µm^2^ fully coalesced structure with a thickness of 3 µm, as shown in Fig. 1 ▸(*b*), and a flat surface (typical roughness is of the order of 1 nm for 5 × 5 µm). A matrix formed by fully coalesced structures is presented in Fig. 1 ▸(*c*), and two fully coalesced matrices are shown in Fig. 1 ▸(*d*) with 1 µm pitch (top) and 0.5 µm pitch (bottom). Some platelets in Fig. 1 ▸(*d*) (bottom) are damaged due to a problem in the pillar fabrication process, and this is the subject of additional research to improve and optimize the pillar etching (Mrad *et al.*, 2022 ▸). Therefore, all measurements in this work were performed only on the good-quality GaN platelets.

Unlike *in situ* techniques, DFXM and the high-intensity diffraction techniques at ESRF are very suitable for studying such complex systems at the nanometric and macrometric levels and understanding the coalescence process. The experiments conducted at the two different beamlines will be presented in the next section.

### 2.2. Characterization techniques

#### 2.2.1. Dark-field X-ray microscopy

**Figure 2:**
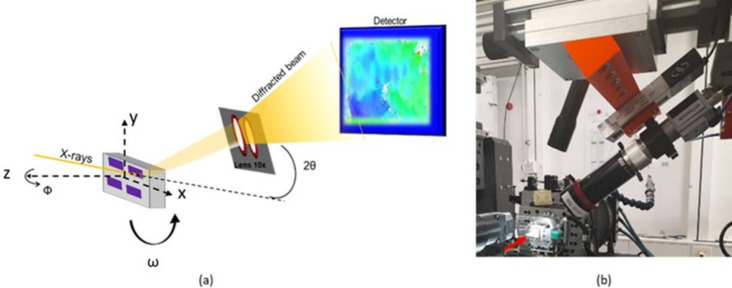
(*a*) A schematic of the DFXM geometry including the rotational axes ω, Φ and 2θ. The gray box is our sample and the purple rectangle represents the GaN coalesced structures. (*b*) A photograph of the instrument at the ID06 beamline at ESRF (the red arrow is pointing at the studied sample).

DFXM, a full field imaging technique, is used to characterize the GaN samples described in Section 1. The whole GaN structure is illuminated with focused monochromatic X-rays. The diffracted beam then passes through a CRL comprising 88 2D Be lenses placed after the sample to produce a 10× magnified inverted real (*x*, *y*) image on the 2D detector. A phi (Φ) or omega (ω) scan can be performed by rotating the sample around its normal axis Φ or the incidence angle ω, as illustrated in Fig. 2 ▸(*a*). These scans provide information on the crystallographic misorientations in the sample, specifically the tilt and twist of the crystallites. We typically performed 40 rocking curves with a step ΔΦ of 0.08° and Δω of 0.02°. A 2θ scan involves rotating the detector and, as it changes the Bragg angle, this gives information on the strain in the material (when combined with an ω scan). A photograph of the experimental setup is shown in Fig. 2 ▸(*b*). The spatial resolution is 100 nm, limited by CRL imperfections, and the beam energy is 16 keV with an energy bandwidth of the order of 1.4 × 10^−4^. For our experiment we used the GaN 101 Bragg reflection to study the GaN structures using a diffraction angle θ = 9.16°.

#### 2.2.2. High-intensity X-ray diffraction

**Figure 3:**
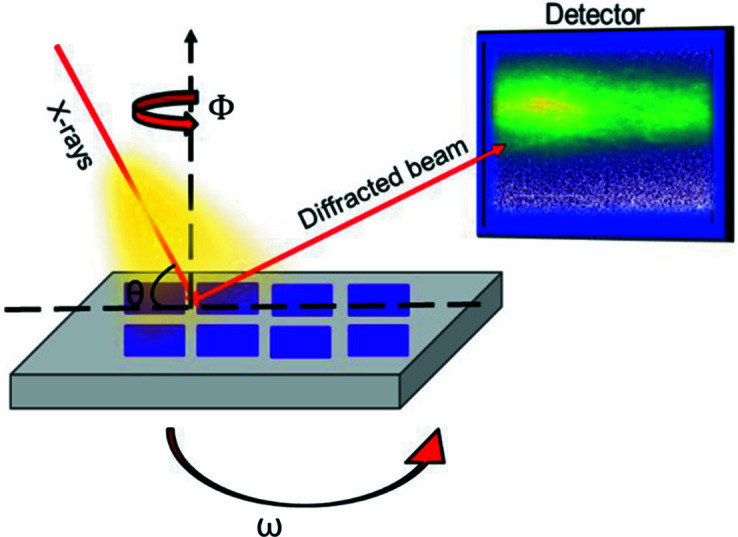
A schematic of the high-intensity X-ray diffraction geometry.

This technique is an X-ray diffraction method that uses a high-brilliance X-ray synchrotron source to measure the structural properties of crystalline matter non-destructively. The source used here is installed on beamline BM02 of ESRF. The energy of the incident beam was fixed at 9 keV with a resolution of 2 × 10^−4^ and an intensity of 2.4 × 10^10^ photons s^−1^. The incident beam arriving at the sample covered an area of two coalesced GaN structures (10 × 10 platelets, 40 × 40 µm^2^ across) (Fig. 3 ▸).

The variation of tilt and twist in the GaN layers was analyzed using the GaN 204 reflection, and the tilt and twist in the Si(111) layer (located between the AlN nucleation layer and the SiO_2_) was analyzed using the Si 111 and Si 331 reflections, respectively. These measurements were carried out for structures with pitch *p* = 0.5 µm and *p* = 1 µm.

## 3. Results and discussion

### 3.1. Dark-field X-ray microscopy

DFXM allowed us to analyze the GaN layer on top of a line of pillars, as well as coalesced GaN structures of 40 × 40 µm^2^, in order to determine their quality and understand the coalescence process.

#### 3.1.1. Line-pattern characterization

**Figure 4:**
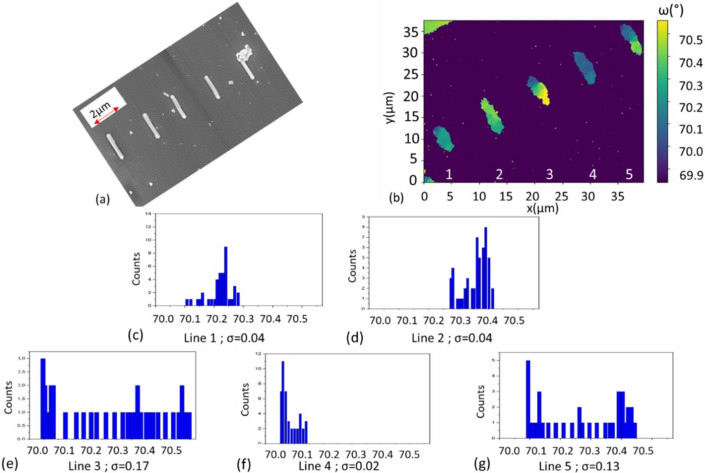
(*a*) A scanning electron microscope image of five fully coalesced GaN lines. (*b*) A COM map for ω of the five lines. The histograms in (*c*), (*d*), (*e*), (*f*) and (*g*) show the distribution of ω along each line (the binning step corresponds to 0.1°).

To understand the process of coalescence in a 2D array of crystallites, we start by studying the coalescence along lines, *i.e.* a 1D array, of ten pillars with a pitch of 0.5 µm. These lines were grown using the same approach and conditions as explained above. However, these conditions were optimized (in particular, the growth time for each step) for the specific 40 × 40 µm^2^ GaN platelets with pitch = 0.5 µm; therefore, they are not necessarily optimal for a single line of pillars even if the pitch is the same. Five fully coalesced lines are seen in the scanning electron microscope image in Fig. 4 ▸(*a*). They were characterized with DFXM using the GaN 101 reflection. The data in Fig. 4 ▸ were obtained from a set of ω–Φ scans and extracted using the *darfix* library (Garriga Ferrer *et al.*, 2022 ▸). Fig. 4 ▸(*b*) shows the center of mass (COM) map for the incidence angle ω obtained for the five lines. The results show significant differences between the lines. For lines 1, 2 and 4, we have homogeneous lines with a very small ω variation along the line itself (Δω < 0, 1°), implying that the GaN crystallites are very well oriented and that few, if any, coalescence boundary dislocations are required to accommodate any misorientation. In contrast, lines 3 and 5 have strong ω variations (Δω > 0, 1°) along their length, implying the generation of dislocations at the interfaces between pillars.

In Figs. 4 ▸(*c*)–4 ▸(*g*), we show histograms of the ω values for each pixel with a Δω of 0.1° for the five lines. The values of the standard deviation (σ) found in lines 1, 2 and 4 are very small, of the order of 0.04°. In lines 3 and 5, we have a much wider range of ω values, and higher σ values of 0.17 and 0.13°, respectively. Kurtz *et al.* (1956 ▸) created a model that relates the full width at half-maximum (FWHM) β of ω scan peaks to the dislocation density and the Burgers vector **b** through equation (1):

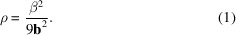

On the basis of this equation, the dislocation density for the well oriented lines (1, 2 and 4) is calculated to be 1.2 × 10^7^ cm^−2^, while previous studies of GaN on Si have shown FWHM values for 002 and 101 reflections of ∼0.13 and 0.19°, respectively (Charles *et al.*, 2018 ▸), corresponding to dislocation densities of the order of 1 × 10^9^ cm^−2^. This shows that, for the good lines (1, 2 and 4), there is a significant improvement. However, lines 3 and 5 are comparable to a standard 2D growth with a dislocation density of 1.7 × 10^9^ cm^−2^, meaning that improvements are still required to better control the growth process.

#### 3.1.2. Coalesced GaN structure characterization

**Figure 5:**
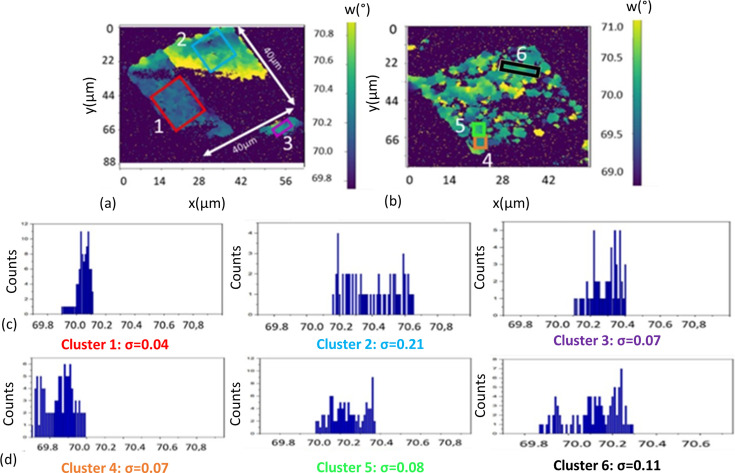
(*a*) A COM map for ω of GaN structure 1 with *p* = 0.5 µm. (*b*) A COM map for ω of GaN structure 2 with *p* = 1 µm. (*c*) Three histograms showing the distribution of ω in clusters 1, 2 and 3 inside structure 1. (*d*) Three histograms showing the distribution of ω along clusters 4, 5 and 6 inside structure 2 (the binning step corresponds to 0.1°).

The results found from the X-ray characterization of lines are promising as they show that it is possible to have excellent alignment of the pyramids during coalescence. The next objective was to investigate the coalesced GaN structure of 40 × 40 µm^2^. Two platelets were analyzed: structure 1 [Fig. 5 ▸(*a*)] with a pitch of 0.5 µm and structure 2 [Fig. 5 ▸(*b*)] with a pitch of 1 µm. Figs. 5 ▸(*a*) and 5 ▸(*b*) show the COMs of the two structures for ω using the GaN 101 reflection from ω–Φ scans. From structure 1, we can see three separate areas. Clusters 1 and 3 in structure 1 are very well oriented areas, with standard deviations (σ) of 0.04 and 0.07°, respectively, as presented in the histograms [Fig. 5 ▸(*c*)]. These values are similar to those found in the homogenous lines discussed above. The resulting dislocation density for cluster 1 is 1.1 × 10^8^ cm^−2^ from equation (1), a value significantly better than standard GaN 2D growth. However, cluster 2 has a higher standard deviation of 0.21°, which is larger than values seen for standard GaN 2D growth.

GaN structure 1 is not a full rectangle due to a parasite nucleation site, whose different orientation prevents it from diffracting at the chosen Bragg angle. This parasite nucleation site originates from growth on top of improperly etched, missing or fractured pillars.

GaN structure 2 has a pitch of 1 µm [Fig. 5 ▸(*b*)], and since the spacing between the pillars is larger, the coalescence is not completely finished. As the coalescence is still ongoing, we can see that it appears to occur through a cluster phenomenon where each group of pillars coalesces to form small areas of well oriented GaN with low standard deviation values, as seen in Fig. 5 ▸(*d*). Clusters 4, 5 and 6 have standard deviation values of 0.07, 0.08 and 0.11°, respectively. Calculations using equation (1) translate these values into dislocation densities of 1.3 × 10^8^, 2.6 × 10^8^ and 9.2 × 10^8^ cm^−2^, respectively.

Although not uniform across the platelets, these results are extremely promising since we are able to achieve high-quality GaN across areas greater than 10 × 10 µm^2^ (structure 1, cluster 1) with our growth approach.

### 3.2. High-intensity X-ray diffraction

High-intensity X-ray diffraction allowed us to analyze macroscopically the platelet samples, and also gave access to the Si(111) layer, which it was not possible to measure using DFXM because the intensity of the diffraction peaks for this thin buried layer is too weak.

#### 3.2.1. Twist and tilt in the GaN layers

**Figure 6:**
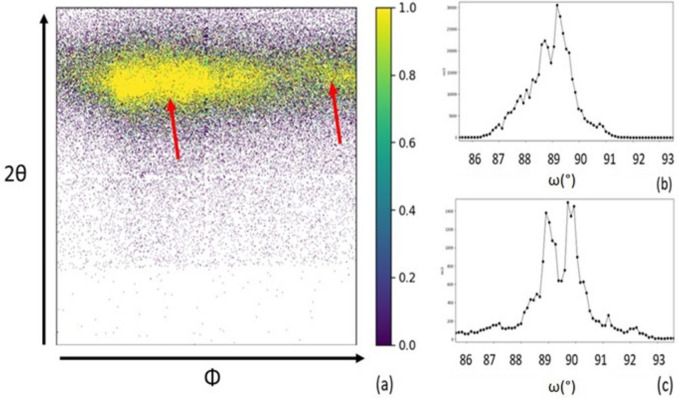
(*a*) A 2D detector image of the GaN 204 Bragg peaks at one position in the GaN structure, with the *x* and *y* axes representing Φ and 2θ, respectively. Intensity is displayed to the right of the image and two Bragg peaks at different values of Φ are shown with the red arrows. (*b*), (*c*) Rocking curves showing two Bragg peaks at different values of ω for (*b*) *p* = 0.5 µm and (*c*) *p* = 1 µm.

Fig. 6 ▸(*a*) shows the diffraction pattern from the 2D detector, with the *y* axis corresponding to values of 2θ and the *x* axis corresponding to Φ. This image is from the GaN 204 Bragg reflection in the middle of a GaN platelet coalesced from pillars with a 0.5 µm pitch. We can clearly see that for a given value of θ and ω we have peaks at different values of Φ, as shown by the red arrows. This implies that we are diffracting from zones or clusters of GaN with different values of twist. In addition, Fig. 6 ▸(*b*) shows the distribution of the intensity as a function of ω for a given Φ value, and we can see the presence of more than one peak at different values of ω: for example, for *p* = 0.5 µm, we identify a peak at ω = 88.8° and another at ω = 89.5°. This also reinforces the cluster detection, but this time with different tilt orientation. Fig. 6 ▸(*c*) shows similar results for the structures coalesced from pillars with a pitch of 1 µm.

These results reinforce the analysis of the growth seen by DFXM, which suggests that coalescence is happening by formation of clusters covering several pillars, which subsequently coalesce as large grains.

By measuring the GaN 204 reflection, we obtained FHWM values of ∼0.6° in the GaN layers of the coalesced structure. This high value can be explained by the fact that with this technique we are scanning across two GaN structures and not just one [Fig. 1 ▸(*b*)]. Thus the results presented correspond to multiple clusters across two structures.

#### 3.2.2. Twist and tilt in the Si(111) layers

To better understand the cluster formation, we also examined the orientation in the Si(111) layers contained within the nano-pillars, beneath the GaN layers.

Our growth approach presented in Section 2.1 assumes a self-alignment of GaN layers during coalescence, which would cause a misorientation of the Si(111) layer at the top of the nano-pillars. To determine the tilt and twist of the nano-pillars induced by coalescence, we measured the asymmetrical Bragg reflection Si 331 and the symmetrical Bragg reflection Si 111 in this top Si(111) layer for two samples. Sample A is a reference sample that has only nano-pillars, *i.e.* prior to the pyramids’ growth. Sample B is composed of the fully coalesced GaN structures described above in Section 2.1, with nano-pillar pitches of 0.5 and 1 µm.

For the 331 reflection, the FWHM of the diffraction curve is broadened in sample B compared with sample A, from 1.1° for sample A to 2.5° for sample B for a pitch of 0.5 µm. It is likely that the initial value of the FWHM is strongly linked to the instrumental resolution and the coherently diffracting domains’ size and shape (Cullity, 1956 ▸). The Si 111 reflection also shows an increase in FWHM between the reference sample A and sample B, going from 0.32° for sample A to 0.81° for sample B for a pitch of 0.5 µm and from 0.25° for sample A to 0.71° for sample B for a pitch of 1 µm.

The broadening of the two diffracted planes implies that the Si layers are more twisted and tilted in the coalesced structures compared with the reference sample, as expected from our model.

## 4. Conclusions and perspective

DFXM and high-intensity X-ray diffraction offer the opportunity to characterize optoelectronic materials at high spatial and angular resolution by examining coalesced lines of GaN pyramids formed on nano-pillars, and 40 × 40 µm^2^ GaN platelets on nano-pillars at both macro- and nano-scale.

At the macroscale, measurements performed by high-intensity X-ray diffraction were beneficial in understanding the coalescence process. Firstly, large peak widths were measured, implying that the coalescence occurs by cluster formation where different clusters have different orientations. Secondly, during coalescence, the Si sections in the nano-pillars become significantly disoriented (compared with a reference sample), which is indirect proof of the self-orientation of the GaN pyramids during the coalescence.

At the nanoscale, with DFXM we showed that we can achieve extremely well oriented lines, with barely any misorientation between pillars along the whole line with our unique growth approach. In addition, when coalesced into platelets, we achieved regions up to 10 × 10 µm^2^ that are also extremely well oriented. These results are extremely promising for the fabrication of small highly oriented islands of GaN on Si, suitable for highly efficient light emission in devices such as micro-displays or micro-LEDs.

The capacities of these powerful techniques open the door to future investigation on newly improved high-quality samples in order to optimize this growth process and improve the understanding of the physical phenomena taking place.

## 5. Data availability

Supporting data associated with this article can be found at https://data.esrf.fr/doi/10.15151/ESRF-ES-819380178 and https://data.esrf.fr/doi/10.15151/ESRF-ES-750661418 for the ID06 and BM02 experiments, respectively.

## Supplementary Material

Supporting data for the ID06 experiment: https://data.esrf.fr/doi/10.15151/ESRF-ES-819380178

Supporting data for the BM02 experiment: https://data.esrf.fr/doi/10.15151/ESRF-ES-750661418
